# Compensation of Hysteresis in the Piezoelectric Nanopositioning Stage under Reciprocating Linear Voltage Based on a Mark-Segmented PI Model

**DOI:** 10.3390/mi11010009

**Published:** 2019-12-19

**Authors:** Dong An, Yixiao Yang, Ying Xu, Meng Shao, Jinyang Shi, Guodong Yue

**Affiliations:** 1 School of Mechanical Engineering, Shenyang Jianzhu University, Shenyang 110168, China; peteryang@stu.sjzu.edu.cn (Y.Y.); xuying@sjzu.edu.cn (Y.X.); mshao@sjzu.edu.cn (M.S.); 2 Research Center for Analysis and Detection Technology, Shenyang Jianzhu University, Shenyang 110168, China; 3 School of Electro-Mechanical Engineering, Guangdong University of Technology, Guangzhou 510006, China; shijy@mail2.gdut.edu.cn

**Keywords:** nanopositioning stage, piezoelectric hysteresis, mark point recognition, piecewise fitting, compensation control

## Abstract

The nanopositioning stage with a piezoelectric driver usually compensates for the nonlinear outer-loop hysteresis characteristic of the piezoelectric effect using the Prandtl–Ishlinskii (PI) model under a single-ring linear voltage, but cannot accurately describe the characteristics of the inner-loop hysteresis under the reciprocating linear voltage. In order to improve the accuracy of the nanopositioning, this study designs a nanopositioning stage with a double-parallel guiding mechanism. On the basis of the classical PI model, the study firstly identifies the hysteresis rate tangent slope mark points, then segments and finally proposes a phenomenological model—the mark-segmented Prandtl–Ishlinskii (MSPI) model. The MSPI model, which is fitted together by each segment, can further improve the fitting accuracy of the outer-loop hysteresis nonlinearity, while describing the inner-loop hysteresis nonlinearity perfectly. The experimental results of the inverse model compensation control show that the MSPI model can achieve 99.6% reciprocating linear voltage inner-loop characteristic accuracy. Compared with the classical PI model, the 81.6% accuracy of the hysteresis loop outer loop is improved.

## 1. Introduction

The nanopositioning stage of the piezoelectric ceramic material driver has the advantages of small volume, high displacement resolution, fast response, large bearing capacity, no noise, and high stability [1]. Hence, it is widely used in modern precision machineries as the core device, such as in atomic force microscopy [2,3] and nanolithography processing [4]. However, the inherent hysteresis nonlinearity of the piezoelectric ceramic materials affects the accuracy of this nanopositioning stage [5]. Thus, it is necessary to model effective compensation for the hysteresis [6,7].

In order to improve the positioning accuracy of the nanopositioning platforms, many scholars at home and abroad have conducted extensive research on piezoelectric ceramic hysteresis. There are two popular approaches. One is to study the hysteresis due to the internal mechanism, where the crystal grains constitute the crystal phase of the piezoelectric ceramic and the electric domains appear in the crystal grains. Polarization treatment enables piezoelectric ceramics to exhibit a piezoelectric effect [8]. However, a small number of grains returns to the original direction after the polarization, which is known as the residual polarization, leading to hysteresis [9]. The second approach is to establish a phenomenological model for the expressed voltage–displacement characteristics, including differential equation models such as the Bouc–Wen model [10], operator models such as the Preisach model [11], the Prandtl–Ishlinskii (PI) model [12,13,14], and others.

Among them, the PI model is weighted and superimposed by the Play operator [15]. The symmetry and hysteresis characteristics of the Play operator can efficiently describe the appearance characteristics of the hysteresis [16]. Compared with the nonlinear integral of the Preisach model and the nonlinear differential equation of the Bouc–Wen model, the PI model has fewer parameters, a simpler structure, and it is easier to find its inverse model. It has been widely used in modeling and compensation of hysteresis features [17,18]. The classical PI model always has symmetry, which makes it hard to accurately approximate the hysteresis characteristics of the boost phase and buck phase at the same time [19].

Many studies have been conducted on how to improve the accuracy of the nanopositioning stage by improving the traditional PI model. There are ways to change the operator by modifying the classical PI model into a generalized PI model, resetting the initial value that can eliminate the influence of the polarization by changing the hysteresis characteristics of the symmetric bias modeling and piecewise fitting method [20,21,22,23]. The above researches greatly improve the hysteresis description accuracy of the nanopositioning stage for single-ring linear voltage.

In a previous study [24], a segmented model was carried out by dividing the boost phase hysteresis characteristics by polarization in single-ring linear voltage. However, it is more likely for an improved method to solve the physical problem than a define-based modeling. For the reciprocating linear voltage hysteresis, the hysteresis characteristics of the inner and outer loops are different and complex. Thus, the segmented modeling method is limited in reciprocating linear voltage due to insufficient theoretical segmentation basis. Therefore, both the classical PI model and the improved model cannot meet the accuracy compensation requirements of the nanopositioning stage under reciprocating linear voltage.

Aiming at the complex hysteresis problem of the piezoelectric stage for the reciprocating linear voltage, this study proposes a mark-segmented PI (MSPI) model that loads the reciprocating linear voltage signal according to the reciprocating linear displacement requirement and compensates using the characteristics of the obtained hysteresis. An approximate hysteresis curve slope can be obtained between the adjacent measurement points of the experimental data, and the segment identification points corresponding to the slope jump segments are found in a threshold manner; thus, the segments are modeled. The results show that the description accuracy of the MSPI model is high, and it has good performance in both the compensation hysteresis inner loop and hysteresis outer loop.

The structure of this paper is as follows. In the second section, a nanopositioning stage with a double-parallel guiding mechanism is designed, and the mechanical characteristics of the platform are analyzed to optimize the design structure. In the third section, the hysteresis characteristics are analyzed and the MSPI model is designed for fitting the reciprocating nonlinear features. After the inversion, the fourth section gives the control comparison between the MSPI model and the classical PI model. Two other verification experiments are also conducted. Finally, the fifth section summarizes this study.

## 2. Precondition

This section is divided into two parts. The first part designs a nanopositioning stage with a double-parallel guiding mechanism, including its mechanical structure selection and stiffness calculation. The second part is the voltage–displacement experiment process and the conclusion obtained.

### 2.1. Mechanical Design and Calculation of Nanopositioning Stage

**Figure 1:**
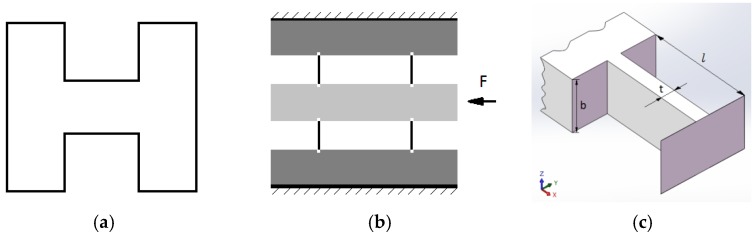
Flexible hinge selection: (**a**) rectangular cut section; (**b**) double-parallel guide mechanism; (**c**) flexible hinge main parameter identification

The nanopositioning stage uses a completely flexible mechanism. This flexible mechanism has a microscale range for the elastic deformation motion. It can achieve the transmission of the motion and force without friction [25,26]. Considering the thinness of the flexible hinge, the wire cutting method is adopted. Therefore, the slit that is easier for the machine is selected as a flexible hinge of a rectangular cross-section, as shown in Figure 1a. The flexible hinge, with four rectangular sections as the base members, is the double-parallel guiding mechanism used in the design, as shown in Figure 1b.

The mechanical model for the rectangular section of the flexible hinge is shown in Figure 1c. Since the movement causes the flexible hinge to elastically deform, the stiffness in each direction must be calculated. According to the equations of material mechanics [27], the stiffness $k_{x}$ and torsional stiffness $k_{\theta x}$ motion in the direction x are:

(1) $$k_{x}=\frac{48EI_{yy}}{l^{3}}=\frac{4Et^{3}b}{l^{3}}$$

(2) $$k_{\theta x}=\frac{Etb^{3}}{l^{3}}B^{2}$$

where E is the modulus of elasticity, t is the thickness of the flexible hinge, b is the width of the flexible hinge, l is the length of the flexible hinge, $I_{yy}$ is the bending section coefficient of the y-axis, and B is the width of the nanopositioning stage.

The stiffness $k_{y}$ and torsional stiffness $k_{\theta y}$ in the vertical direction y are:

(3) $$k_{y}=\frac{12EI_{zz}}{l^{3}}=\frac{Etb^{3}}{l^{3}}$$

(4) $$k_{\theta y}=\frac{Etb}{l}D^{2}$$

where $I_{zz}$ is the bending section coefficient of the z-axis and D is the distance between the flexible hinges of the two parallel rectangular sections.

The stiffness $k_{z}$ and torsional stiffness $k_{\theta z}$ in the vertical direction *z* are:

(5) $$k_{z}=\frac{4EA}{l}=\frac{Etb}{l}$$

(6) $$k_{\theta z}=\frac{Etb^{3}}{3l}\left(\frac{D^{2}}{2l^{2}}+\frac{t^{2}}{5b^{2}}\right)$$

where A is the flexible hinge cross-sectional area.

The designed flexible hinge has a length of 14.5 mm, a thickness of 0.3 mm, a width of 15 mm, and a material elastic modulus of 72 GPa. The maximum equivalent stress is 7.8 MPa. The stiffness of the double-parallel guiding positioning platform at six degrees of freedom is obtained, as shown in Table 1.

**Figure 2:**
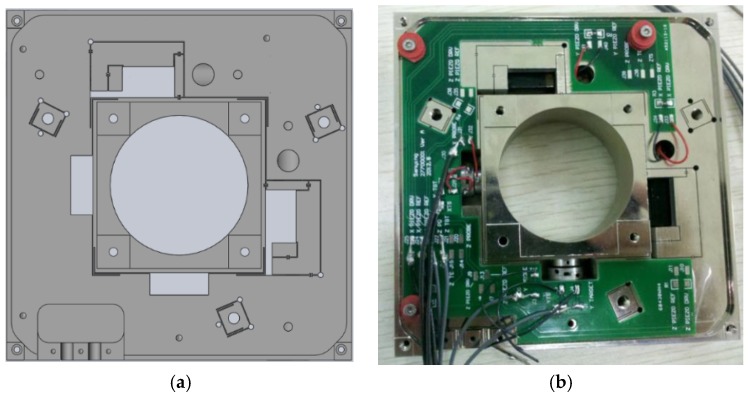
Double-parallel guiding mechanism nanopositioning stage: (**a**) the schematic diagram (**b**) and the actual diagram.

In order to strictly guarantee the accuracy of the nanopositioning stage, a microlevel precision slow wire cutting technology is adopted [28]. At the same time, a material with a small thermal expansion coefficient is selected [29]. In order to prevent the wire mechanism from oxidizing, the surface of the flexible hinge needs to be nickel plated. Figure 2a shows a schematic diagram of the nanopositioning stage. Figure 2b is the actual diagram of the nanopostioning stage with double-parallel guiding mechanism.

### 2.2. Test Results

**Figure 3:**
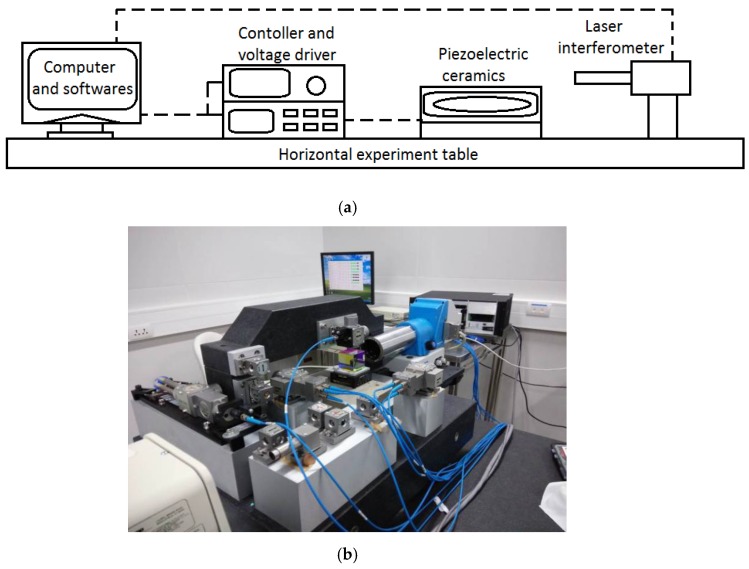
Nanopositioning stage experimental system: (**a**) system schematic (**b**) and actual system diagram.

The experimental system was built using a laser interferometer, nanopositioning platform, reflection mirror, controller, and computer software, as shown in Figure 3a. The laser interferometer used is the Renishaw XL-80 series achieves an accuracy of ± 0.5 ppm. The selected driver was the HVA-150D.A3 instrument from Harbin Xinmingtian Company, whose voltage input variable range is 0 V-150 V. Figure 3b shows the actual experimental system.

**Figure 4:**
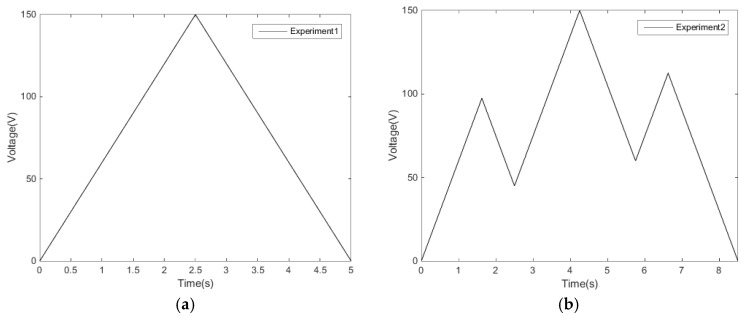
Experimental loading voltage: (**a**) single-ring linear voltage (**b**) and reciprocating linear voltage.

The user performs the following steps to test the output displacement characteristics of the nanopositioning stage in the x-direction motion, under single-ring linear voltage and under reciprocating linear voltage:

(1) Adjust the laser interferometer so that its light signal intensity is within the confidence range. Connect the computer, controller, and laser interferometer via the USB interface data cable. Data is collected on the computer by the corresponding software of the controller and laser interferometer.

(2) The controller matching software loads the electrical signal to the driving controller. The initial driving voltage is 0 V. Experiment 1 is carried out to give a linear voltage rise signal from 0 V to 150 V. The displacement is measured and recorded by laser interferometer for every 7.5 V. Next, a linear voltage reduction signal is sent from 150 V to 0 V. The displacement is measured and recorded every 7.5 V. A single-ring linear voltage signal is shown in Figure 4a.

(3) Similarly, experiment 2 is carried out. The reciprocating linear voltage signal is recorded, as shown in Figure 4b.

(4) Record several measurements.

(5) Check the instrument and turn it off. Process experimental data.

**Figure 5:**
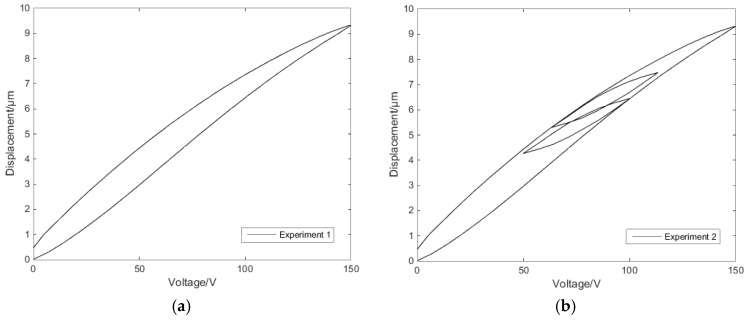
Voltage–displacement experimental curves: (**a**) single-ring linear voltage (**b**) and reciprocating linear voltage.

Through the above experiment, two sets of data can be obtained: a single-ring linear voltage–displacement characteristic curve, as shown in Figure 5a; and a reciprocating linear voltage–displacement characteristic curve, as shown in Figure 5b.

The expected voltage displacement curves should be linear. Analysis of the experimental data established the nonlinearity of the experimental curve. The voltage displacement curve at the single-ring linear voltage has a hysteresis characteristic. The voltage displacement curve at the reciprocating linear voltage has the same hysteresis characteristics as the single-ring voltage’s, while the reciprocating voltage’s hysteresis loop conforms to the Madelung principle [30]. Therefore, in order to solve the hysteresis characteristics of the piezoelectric ceramic under single-ring linear voltage and reciprocating linear voltage, an effective compensation method is needed.

## 3. Modeling

This section describes the process of establishing the MSPI model in three parts. In the first, part the curve of the classical PI model is obtained to describe the hysteresis characteristics. The second part analyzes the specific problem of the classical PI model’s inner-loop hysteresis description, and defines the voltage–slope curve corresponding to the hysteresis rate tangent to establish the MSPI model. The third part uses a threshold method to judge whether it is the mark point. The segmentation of the mark points gives the curve described by the MSPI model and its inverse model.

### 3.1. Play Operator and Classical Prandtl–Ishlinskii Model

**Figure 6:**
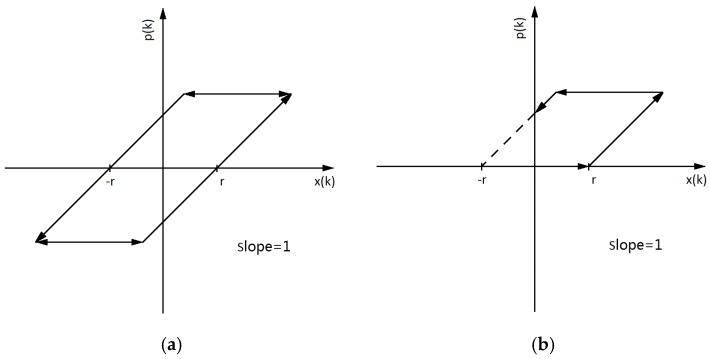
Play operator models: (**a**) full-sided operator model (**b**) and single-sided operator model.

The classical PI model is a weighted superposition of a finite number of Play operators. The Play operator is shown in Figure 6a. When the input signal is x(k), the Play operator expression with the threshold r is:

(7) p(k)=max{x(k)−r,min[x(k)+r,p(k−1)]}

where $0=k_{0}<k_{1}<\ldots <k_{s}$ is the appropriate division on the input signal interval, $k\in \left[0,k_{s}\right]$. When k=0, p(−1) is the initial value. In the PI model of the piezoelectric effect hysteresis problem, the initial voltage is usually 0 without displacement, so p(−1)=0. Here, p(k) is the output of the input signal.

The voltage supplied by the voltage driver is positive, and hence the PI model is usually modeled with a single-sided Play operator. As shown in Figure 6b, when the operator inputs x(k)≤r, the operator outputs p(k)=0; when the operator inputs $r<x(k)\leq x(k_{s})$, the unweighted operator has a slope of 1, so the operator outputs p(k)=x(k)−r. The operator shown in Figure 6b outputs $p(k)=x(k_{s})$ when the input decreases from x(k) to $x(k_{s})-2r$ and outputs p(k)=x(k)+r when the input decreases from $x(k_{s})-2r$ to 0. The operator may have no p(k)=x(k)+r output and a part of $p(k)=x(k_{s})$, when the threshold r is increased or the input $x(k_{s})$ is decreased. In this case, the specific characteristics of the operator should be considered.

A finite number of Play operators are superimposed according to the weighting of the above output characteristics, and a PI model is obtained to describe the hysteresis of the nanopositioning stage. The equation is:

(8) $$P\left[x(k)\right]=\theta_{0}\cdot x(k)+\sum_{i=1}^{n}\theta_{i}\cdot p_{i}(k)$$

where P[x(k)] is the corresponding PI model output for the operator input x(k). Here, $\theta_{0}$ is a positive value, $p_{i}(k)$ is the output of the ith operator that has a threshold $r_{i}$ and a corresponding weight $\theta_{i}$.

The more times the PI model is superimposed, the smoother the model contour is and the closer it is to the piezoelectric hysteresis characteristic curve. However, the accuracy of the voltage–displacement characteristics obtained from the experiments is limited, so the number of operators used for the superposition should be realistic.

**Figure 7:**
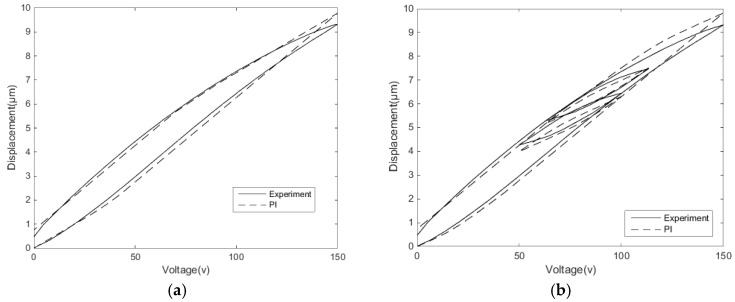
PI modeling: (**a**) modeling of single-ring linear voltage hysteresis characteristics and (**b**) modeling of reciprocating linear voltage hysteresis characteristics.

Figure 7 shows the modelling of the PI model to display the single-ring linear voltage hysteresis characteristic and reciprocating linear voltage hysteresis characteristic of the second section. The modeling results show that the classical PI model describes the hysteresis characteristics well under single-ring linear voltage, but the accuracy under the reciprocating linear voltage is comparatively poor. The main reason is that the hysteresis characteristics of the reciprocating linear voltage are more complicated, and the hysteresis rates between the inner loop and the outer loop are different. Meanwhile, the classical PI model cannot describe the Madelung principle of the hysteresis inner loops.

### 3.2. Hysteresis Tangent Line and Slope

**Figure 8:**
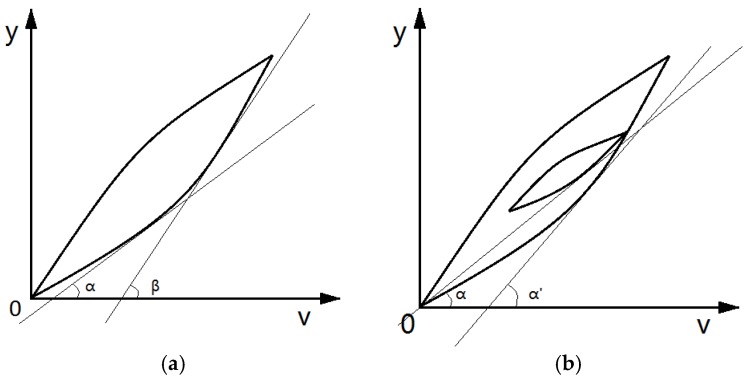
Tangent of the hysteresis loop: (**a**) the hysteresis rate corresponding to each point on the same hysteresis loop is different; (**b**) the hysteresis rates between the inner loop and outer loop of the hysteresis are different. Note: v = voltage; y = displacement.

In order to further improve the description accuracy of hysteresis characteristics, the hysteresis rates must be studied in depth. The hysteresis rates corresponding to the voltage–displacement characteristic curve are the weighted superposition of the Play operators in the PI model at that point. The weight $\theta_{i}$ of the ith operator depends on the angle α between the tangent line of the hysteresis loop at that point and the v-axis. The angle α, as shown in Figure 8a, is not exactly the same in each tangent on the hysteresis loop. The hysteresis rate of the reciprocating linear voltage is even more complicated. As shown in Figure 8b, the tangent at approximately similar positions of the inner loop and outer loop tends to have different hysteresis rates.

The voltage–displacement data can approximate the characteristic curve, thereby establishing a v–y coordinate system. If j is the jth data obtained by the experiment, the hysteresis loop passes through the point $\left(v_{j},y_{j}\right)$. The equation for the hysteresis rate tangent $l_{\tan}(v)$ at v is defined as:

(9) $$l_{\tan}(v):y=s(v)\cdot v+t(v)$$

where s(v) is the hysteresis rate tangent slope at v and t(v) is the hysteresis rate’s tangent intercept at v.

**Figure 9:**
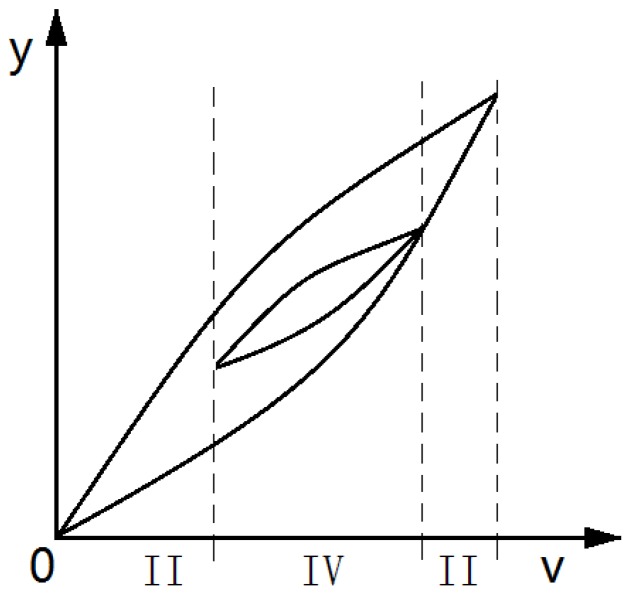
The voltage value corresponding to hysteresis characteristics has more than one tangent. Note: v = voltage; y = displacement.

In a single-ring linear voltage hysteresis characteristic curve, the voltage v corresponds to two hysteresis tangent lines in the linear boost phase and the linear back phase, respectively. Similarly, in the reciprocating linear voltage hysteresis characteristic curve, the v value is likely to correspond to a plurality of hysteresis tangent lines; for example, the hysteresis rate tangent number in Figure 8b corresponding to v is as shown in Figure 9.

The slope of the hysteresis tangent can reflect the trend of hysteresis at this point. The hysteresis tangent slope s(v) can be expressed as:

(10) $$s(v)=\frac{\delta y}{\delta v}=\frac{y_{j+1}-y_{j}}{v_{j+1}-v_{j}}$$

where $\left(v_{j},y_{j}\right)$ and $\left(v_{j+1},y_{j+1}\right)$ are adjacent data and satisfy the equation $\min(v_{j},v_{j+1})\leq v<\max(v_{j},v_{j+1})$.

**Figure 10:**
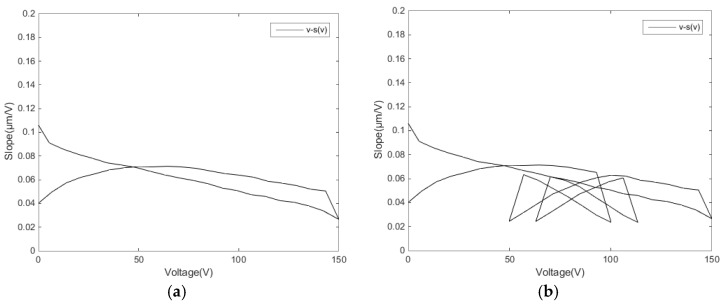
Voltage hysteresis rate tangent slope diagrams: (**a**) single-ring linear voltage and (**b**) reciprocating linear voltage.

The voltage–slope diagram describes the characteristics of the hysteresis rate at any voltage. The different input linear voltage leads to varied hysteresis rate tangent regulation. Figure 10 is the v−s(v) diagram of experimental data for the single-ring linear voltage and the reciprocating linear voltage, respectively. Both groups of data evidently show segmentation in the v−s(v) diagram.

Due to the fact that the piezoelectric hysteresis characteristic generally has a segmentation variation rule and there are obvious jump points between the segments, a segmented PI model is used to model it.

### 3.3. Mark-Segmented Prandtl–Ishlinskii Model

The voltage–slope diagram embodies the change of the hysteresis rate. For reciprocating hysteresis, plenty of turning points appear at the critical edge of boost phases and back phases. Compared with the hysteresis under single-ring linear voltage [24], reasonable identification I required for all mark points in order to fulfill the demands of complex hysteresis segmentation. Meanwhile, data with continuous and similar variation laws should be modeled in the same segment. Therefore, a mark-segmented PI (MSPI) model is proposed.

To identify the segmentation mark point, the threshold φ is set in the v−s(v) diagram. The threshold φ is directly proportional to the quantity of experimental data, which of the minimum data amount is always 8–10 times of the average data difference. When the mth hysteresis rate tangent slope value segment satisfies $s(v_{m+1})-s(v_{m})$, then $|s(v_{m+1})-s(v_{m})|\geq$ φ; $s(v_{m+1})-s(v_{m})$ is defined as the hysteresis rate jump segment. Data $\left(v_{m+1},y_{m+1}\right)$ is defined as the type I mark point.

**Figure 11:**
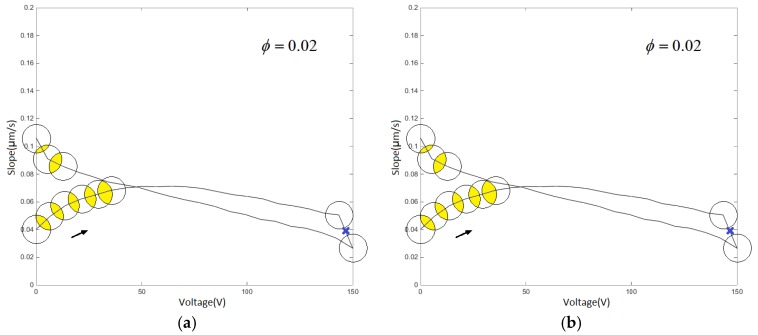
Type I mark points associated with threshold φ: (**a**) single-ring linear voltage v−s(v) characteristic diagram and (**b**) reciprocating linear voltage v−s(v) characteristic diagram.

Therefore, the single-ring linear voltage characteristic experimental curve can be segmented to find one type I mark point, which divides the hysteresis characteristic into 2 segments, as shown in Figure 11a; the reciprocating linear voltage characteristic experimental curve finds five type I mark points, and the data is divided into 6 segments, as shown in Figure 11b.

**Figure 12:**
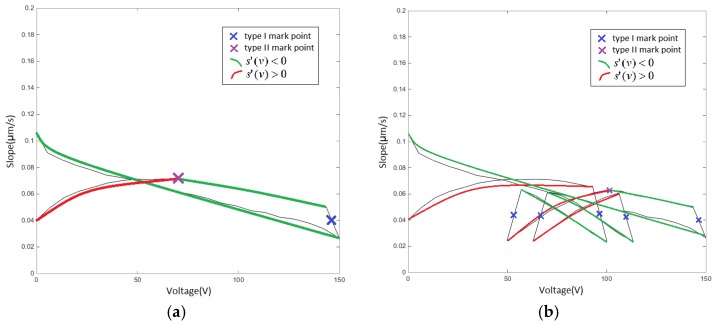
Type II mark point associated with s′(v): (**a**) single-ring linear voltage v−s(v) diagram and (**b**) reciprocating linear voltage v−s(v) diagram.

The segmentation data is required to select an appropriate single-sided Play operator according to its approximate hysteresis characteristics or according to the concavity and convexity. In most cases, the condition for selecting the single-sided Play operator satisfies s′(v)<0 or s′(v)>0,where s′(v) is the differential coefficient of s(v). If there are still some cases where the segmentation data satisfies both the abovementioned conditions at the same time, then it needs to be divided by the segmentation marker point at s′(v)=0, which is defined as a type II mark point. The v−s(v) diagram obtained from the experimental data is not derivable, and the maximum or minimum value can be used as the type II mark point. In the single-ring linear voltage hysteresis v−s(v) diagram shown in Figure 12a, one type II mark point is found, and a total of two segmentation mark points divide the curve into three segments. The reciprocating linear voltage hysteresis characteristic v−s(v) diagram shown in Figure 12b finds one type II mark point, and the total number of segments is 7. Eventually, each segment selects a single-sided Play operator by characteristics.

**Figure 13:**
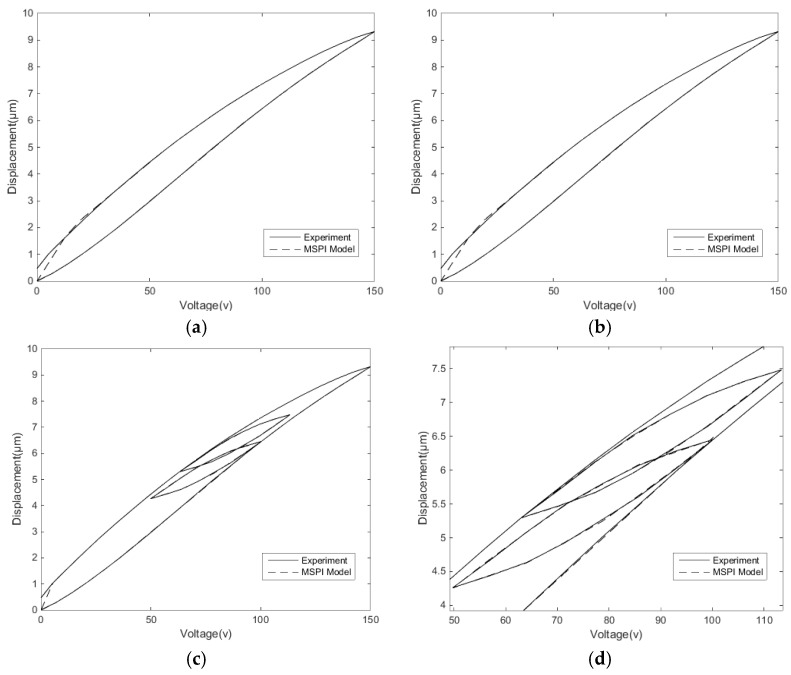
The mark-segemented Prandtl–Ishlinskii (MSPI) model: (**a**) modeling of single-ring linear voltage hysteresis; (**b**) modeling diagram (a) of partial amplification; (**c**) modeling of reciprocating linear voltage hysteresis characteristics; (**d**) modeling diagram (c) of partial amplification.

The placement of the segmentation points is special because they participate in the modeling in both the segments that are divided by themselves. As shown in Figure 13a, the two segmentation points participate in the fitting of the three segments. The MSPI model with single-ring linear voltage hysteresis has good connectivity at the segmentation point. Figure 13b amplifies the MSPI model at one of the mark-segmented points.

Similarly, the five segmentation points of the MSPI model of the reciprocating linear voltage shown in Figure 13c participate in the fitting of the six segments. The hysteresis inner-loop MSPI model of the reciprocating linear voltage is enlarged and shown in Figure 13d. The inner-loop hysteresis characteristic can hence be accurately described.

During the modeling process, the slope of the MSPI model at the end is often larger than the tangent slope of the hysteresis rate that is caused by the forced zeroing of the end of the Play operator. This problem can be solved by ignoring the self-property of the superposition end, and by adding end segmentation and modeling according to its specific hysteresis characteristics.

### 3.4. Inverse Control

**Figure 14:**
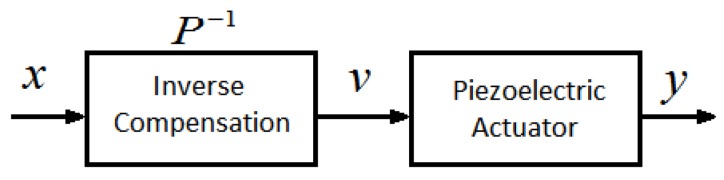
Hysteresis compensation control system diagram.

The MSPI model obtains an accurate approximation of the voltage–displacement correspondence. In order to achieve accurate compensation of the linear displacement, the displacement–voltage correspondence of the MSPI inverse model is used as a feedforward control. According to the compensation control principle [31], the MSPI inverse model is the inverse function of the hysteresis characteristic curve, as shown in Figure 14.

The classical PI model has an analytical inverse. The MSPI model is composed of separate PI models, so the inverse model equation is consistent with the PI inverse model. The equation is:

(11) $$\begin{array}{l} P^{-1}\left[p(k)\right]=\theta_{0}\prime \cdot p(k)+\sum_{i=1}^{n}\theta_{i}\prime \cdot x_{i}(k)=\theta_{0}\prime \cdot p(k)+\\ \sum_{i=1}^{n}\theta_{i}\prime \cdot \max\left\{p(k)-r_{i}\prime ,\min\left[p(k)+r_{i}\prime ,x_{i}(k-1)\right]\right\} \end{array}$$

where $P^{-1}\left[p(k)\right]$ is the output corresponding to the PI inverse model operator input p(k). Here, $x_{i}(k)$ is the output of the ith operator, $\theta_{0}\prime =\frac{1}{\theta_{0}}$. The threshold and weight coefficient of the inverse model are:

(12) $$r_{i}\prime =\sum_{h=1}^{i}\theta_{h}\cdot \left(r_{i}-r_{h}\right)\;\;i=1,2,\cdots ,n$$

(13) $$\theta_{i}\prime =-\frac{\theta_{i}}{\left(\theta_{0}+\sum_{h=1}^{i}\theta_{h}\right)\left(\theta_{0}+\sum_{h=1}^{i-1}\theta_{h}\right)}\;\;i=1,2,\cdots ,n-1$$

**Figure 15:**
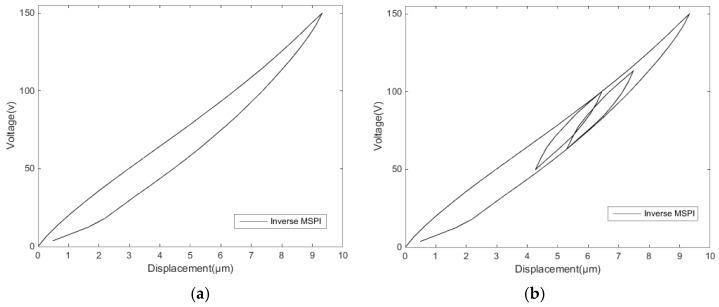
The MSPI inverse models: (**a**) single-ring linear voltage hysteresis inverse model and (**b**) reciprocating linear voltage hysteresis inverse model.

Figure 15 is an MSPI inverse model corresponding to Figure 13a,c. It can be seen that the MSPI inverse model has ideal connectivity between the segments.

## 4. Results

This section presents the experimental results of this study. In the first part of this section, the two typical examples of the MSPI model presented in the third section of this study, namely the single-ring linear voltage hysteresis and the reciprocating linear voltage hysteresis, whose compensation control voltages are obtained by the inverse models, are verified and the errors are analyzed. The second part of this section carries out two verification experiments. One of them studies the effects of different frequency voltages on the MSPI model. The other one tests the MSPI model for another type of nanopositioning stage and observes the modeling effect. All of the above experiments demonstrate the contribution of the MSPI model to improving the nanopositioning accuracy of the stage.

### 4.1. Compensation Results

In order to verify the compensation control effect of the MSPI inverse model, the following experiment was performed on the experimental system from Figure 3, with the inverse model as an input:

(1) Adjust the laser interferometer. Connect computer, controller, and laser interferometer. The related software is turned on and waits for the measurements.

(2) Use the controller-related software to load the control voltage in the inverse model. Experiment 1 is carried out according to the voltage obtained by inverse model of Figure 15a, and the displacement is measured and recorded by the laser interferometer.

(3) Perform the experiment according to the voltage obtained by the inverse model in Figure 15b. Measure and record the displacement data by the laser interferometer. The interval should be the same as (2).

(4) Take several measurements.

(5) Check the equipment and turn it off. Process the experimental data.

**Figure 16:**
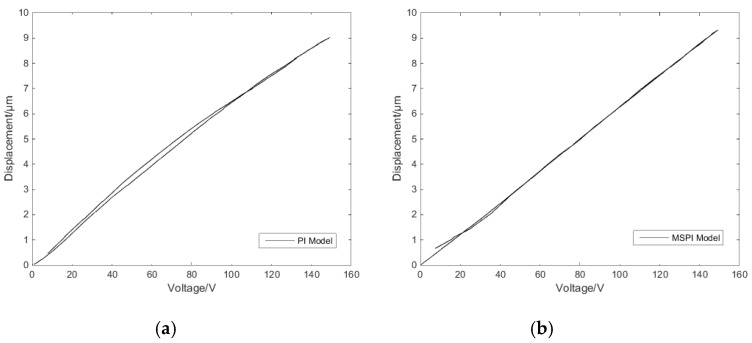
Single-ring linear voltage hysteresis compensation control effects: (**a**) classical Prandtl–Ishlinskii (PI) inverse model compensation and (**b**) MSPI inverse model compensation.

Figure 16a is the measured single-ring linear voltage hysteresis feature compensation effect, while Figure 16b is the hysteresis compensation effect of the classical PI inverse model. The mean absolute deviation can be expressed as:

(14) $$e=\frac{1}{\eta }\sum_{\mu =1}^{\eta }(\varepsilon_{\mu }-\omega_{\mu })$$

where η is data quantity, $\varepsilon_{\mu }$ are expected results, and $\omega_{\mu }$ are experimental results. Hence, the mean absolute deviation of the classical PI inverse model compensation control is 190.2 nm, the mean absolute deviation of the MSPI inverse model compensation control is 35.0 nm, and the nanopositioning accuracy is improved by 81.6%.

**Figure 17:**
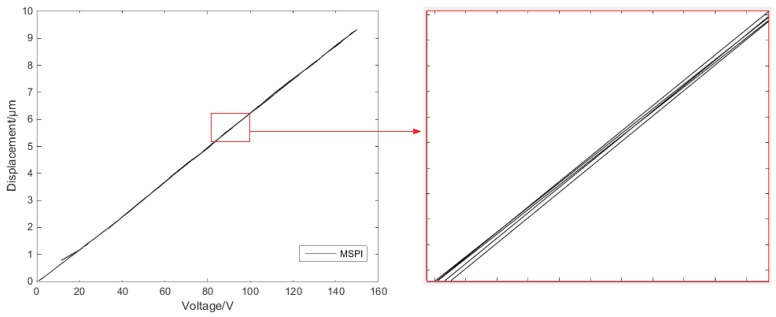
Reciprocating linear voltage hysteresis compensation control effect.

The reciprocating linear voltage hysteresis feature compensation effect is shown in Figure 17. The MSPI model in Figure 13c describes the hysteresis characteristics significantly better than the classical PI model description in Figure 7b; hence, the comparison is not made here. The mean absolute deviation of the MSPI inverse model compensation control of reciprocating linear hysteresis is 19.7 nm, and the positioning error is only 0.42%.

As predicted, the MSPI model is still flawed in its description of the end curve. Therefore, whether it is the reciprocating linear voltage hysteresis MSPI inverse model or the single-ring linear voltage hysteresis MSPI inverse model, the most significant error for both models is at the end of the compensation result. Accuracy can be improved without the end error or with additional segmentation modeling at the end.

In addition, in the two given examples, the MSPI model has more advantages when solving reciprocating linear voltage hysteresis compensation. Compared with the single-ring linear voltage hysteresis MSPI inverse model, the reciprocating linear voltage hysteresis MSPI inverse model has more marks and more segments, and hence the accuracy is improved by 43.7%.

### 4.2. Verification Tests

In order to study whether the change of the voltage frequency affects the application of the MSPI model, verification experiment 1 is carried out to observe the hysteresis characteristics.

For example, it is observed that for single-ring linear voltage, the elongation speed of the piezoelectric ceramic displacement is a negative value and the shrinkage speed is a positive value. The amplitude of the triangular wave voltage is set to be the same as the amplitude of Figure 4a, which is 150V. Three sets of the speed time diagrams can be obtained by changing the voltage frequency. According to the period–frequency relationship $T=\frac{1}{f}$, the smaller the frequency is, the longer the triangular wave voltage period. The appropriate and easily observed frequency control period time is between 1 and 5 s.

**Figure 18:**
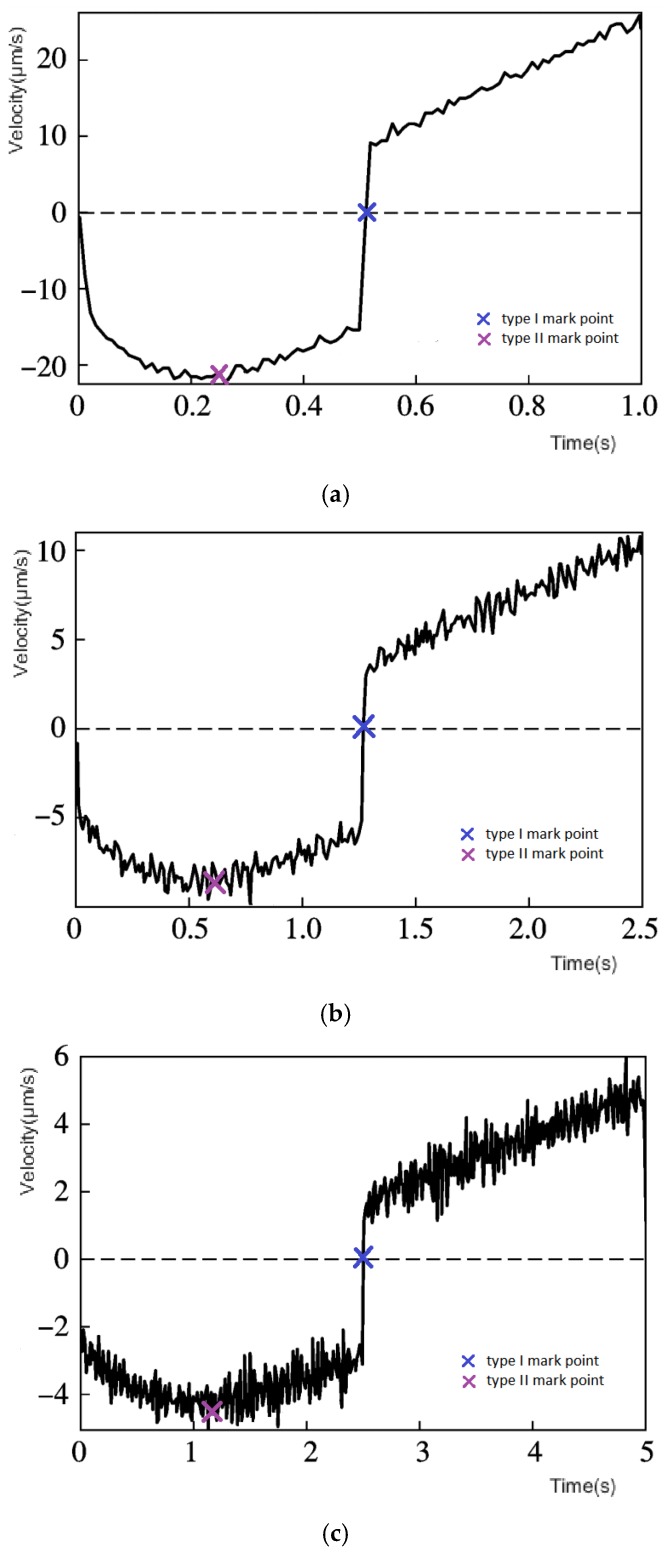
Characteristics of displacement velocity at different frequencies: (**a**) amplitude 150 V, frequency 1.0 Hz; (**b**) amplitude 150 V, frequency 0.4 Hz; (**c**) amplitude 150 V, frequency 0.2 Hz.

Figure 18 shows the speed time diagrams of 1.0 Hz, 0.4 Hz, and 0.2 Hz voltage frequencies.

Although the output speeds are different at different frequencies and the maximum displacement time is shortened as the frequency increases, the same variation characteristics are maintained. If the speed is taken as an absolute value, the speed–time diagrams can find a similar relationship as with the v−s(v) diagram. Both the type I mark points and the type II mark points have been identified on the map in the same color as the v−s(v) diagram.

The hysteresis characteristic of the reciprocating linear voltage is the same. It can be seen that the different voltage frequencies exhibit the same regularity for hysteresis characteristics, and thus the MSPI model is still effective.

Verification experiment 2 is then carried out. The MSPI model for another type of nanopositioning stage is used to observe the modeling effect.

The equipment is adjusted and connected according to the experimental steps in Section 2.2. The triangular wave voltages of 20 V and 15 V amplitudes are loaded as the reciprocating linear voltage inputs. The experimental data are measured and recorded for every 0.5 V.

**Figure 19:**
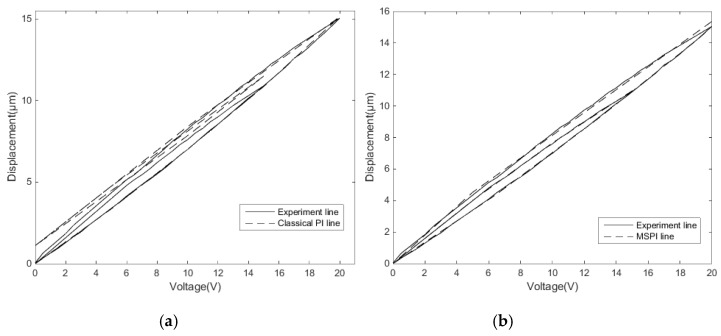
Comparison of the experimental measurements: (**a**) the classical PI model and (**b**) the MSPI model.

Figure 19 shows the hysteresis characteristics of the experimental measurements and the modeling comparison between the classical PI model and MSPI model. The MSPI model has a higher description accuracy, which repeatedly proves that the MSPI model can be applied to different hysteresis characteristics.

## 5. Conclusions

The MSPI model effectively solves the problem of low description accuracy of reciprocating linear voltage hysteresis of the classical PI model. In this paper, the experimental data can be used to find the hysteresis regulation when the hysteresis rate tangent is defined. With a defined segmented basis, the slope characteristics are analyzed to propose a mark-segmented point, which carries out a theoretical solution to segmented modeling. It has been verified that the further segmented modeling can not only compensate for the nonlinear characteristics of the external hysteresis loop under various linear voltages, but can also effectively identify the segmentation of features generated by the intrinsic microscopic mechanism.

The MSPI model hysteresis compensation method does not introduce a new operator. The hysteresis characteristic parameters are completely based on the experimental data. The two types of mark-segmented point recognition methods are simple and evident. Hence, the segmented basis makes the MSPI model reliable. The model construction is easy to implement as well. This paper also provides sufficient theoretical preparation for further study of the more complex hysteresis characteristics of the nanopositioning stage under the nonlinear voltage.

## Figures and Tables

**Table 1:** Stiffness of double-parallel-oriented positioning platform under six degrees of freedom (unit: $k_{x}$, $k_{y}$, $k_{z}$: N/mm; $k_{\theta x}$, $k_{\theta y}$, $k_{\theta z}$: N·mm/rad).

Stiffness	$k_{x}$	$k_{y}$	$k_{z}$	$k_{\theta x}$	$k_{\theta y}$	$k_{\theta z}$
Theoretical value	68.5	99,009.9	22,630.6	21,835,421	100,093,750	7,911,302.6
Calculated value	64.0	106,110.0	29,392.0	23,629,000	107,800,000	7,089,400.0
Error	−6.6%	7.2%	29.9%	8.2%	7.7%	−10.4%
